# Cow’s milk allergy in Dutch children: an epigenetic pilot survey

**DOI:** 10.1186/s13601-016-0105-z

**Published:** 2016-05-04

**Authors:** Nicole C. M. Petrus, Peter Henneman, Andrea Venema, Adri Mul, Femke van Sinderen, Martin Haagmans, Olaf Mook, Raoul C. Hennekam, Aline B. Sprikkelman, Marcel Mannens

**Affiliations:** Department of Pediatric Respiratory Medicine and Allergy, H7-270, Emma Children’s Hospital Academic Medical Center, Meibergdreef 9, 1105 AZ Amsterdam, The Netherlands; Department of Clinical Genetics, DNA-Diagnostics Laboratory, Amsterdam Medical Center, Amsterdam, The Netherlands

**Keywords:** Cow’s milk allergy, Epigenetics, Food allergy, Gender differences, Tolerant

## Abstract

**Background:**

Cow’s milk allergy (CMA) is a common disease in infancy. Early environmental factors are likely to contribute to CMA. It is known that epigenetic gene regulation can be altered by environmental factors. We have set up a proof of concept study, aiming to detect epigenetic associations specific with CMA.

**Methods:**

We studied children from the Dutch EuroPrevall birth cohort study (N = 20 CMA, N = 23 controls, N = 10 tolerant boys), age and gender matched. CMA was challenge proven. Bisulfite converted DNA (blood) was analyzed using the 450K infinium DNA-methylation array. Four groups (combined, girls, boys and tolerant boys) were analysed between CMA and controls. Statistical analysis and pathway-analysis were performed in “R” using IMA, Minfi and the global-test package. Differentially methylated regions in *DHX58*, *ZNF281*, *EIF42A and HTRA2* genes were validated by quantitative amplicon sequencing (ROCHE 454^®^).

**Results:**

General hypermethylation was found in the CMA group compared to control children, while this effect was absent in the tolerant group. Methylation differences were, among others, found in regions of *DHX58*, *ZNF281*, *EIF42A and HTRA2* genes. Several of these genes are known to be involved in immunological pathways and associated with other allergies.

**Conclusion:**

We show that epigenetic associations are involved in CMA. Although, the statistical power of our study is limited and our sample was based on whole blood, we were still able to detect feasible loci and pathways. Therefore our findings might contribute to future diagnostic or therapeutic interventions for specific CMA. Further studies have to confirm the findings of our study.

**Electronic supplementary material:**

The online version of this article (doi:10.1186/s13601-016-0105-z) contains supplementary material, which is available to authorized users.

## Background

### Cow’s milk allergy

Cow’s milk allergy (CMA) is a common food allergy in young children. Accurate data on the worldwide incidences on CMA is still lacking because of discrepancies between self-reported and proper diagnosed allergy. A recent European study describes an incidence of CMA of 0.74 % [1–3]. CMA has a heterogeneous clinical presentation, with a low to moderate estimated heritability of 15 % compared to other (food) allergies [4, 5]. Accumulating evidence in the development of allergic diseases suggest involvement of different pre- and postnatal environmental factors, like gut-microbiota, maturation of the immune-system and epicutaneous allergen sensitization, but also a parent-of-origin factor [4, 6–8]. Young children are likely to develop tolerance for cow’s milk protein (CMP) within a few years [4, 9]. However, infants who suffered from CMA in their early childhood have an increased risk to develop other allergic diseases like asthma later in life, the so-called allergic march [9–13].

### (Epi)genetic component of food allergy

Complex diseases are characterized by a considerable heterogenic phenotype, genetic aberrations but also include gene-environment interactions. Reports on heritability estimations for food allergy (FA) range from 15 % for milk to 82 % for peanut allergy [5, 14]. Some of these reported heritability estimates differ largely between study populations. Whether this is due to study design, geographic or ethnic background or involvement of different environmental factors is not clear [7, 14]. Several environmental components like maternal lifestyle, diet, stress and hygiene, have been suggested to be involved in food allergies [14–18]. Furthermore, accumulating evidence suggest the prevalence of FA in well-developed countries has been increasing over the last decades. Whether this increase is truly occurring is still under debate due to limited amount of comparative data. [19, 20] Genetic selection or genetic drift both are relative slow processes and generally occur in isolated populations, therefore it is highly unlikely that the suggested increase of FA is caused by genetic changes or drift within these populations. These observations favour the possibility that an epigenetic component is involved in the expression of FA and CMA.

### Allergy with respect to gender

The role of gender and sex hormones in relation to the expression of allergy has been studied in epidemiological surveys. The distribution between genders is greatly skewed with regard to the expression of asthma before and after puberty [21, 22]. In general, allergy is more prevalent in boys before puberty, while after puberty allergies are more prevalent in girls. Reports on differences between genders suffering from specific food allergies are however inconsistent [6]. Especially, the studies based on self-reported allergy questionnaires and studies based on a food challenge (the gold standard) show different outcomes [6]. For CMA, differences in prevalence between genders are still under debate [23, 24].

In this pilot case–control study, we investigated if (1) epigenetic differences can be shown between children with proven CMA and healthy controls, (2) if epigenetic differences alter upon obtaining cow’s milk protein tolerance and (3) whether epigenetic differences are reflected by phenotypic differences with regard to gender in the CMA population.

## Methods

### Dutch EuroPrevall birth cohort study

All children were participating in the Dutch EuroPrevall birth cohort study. The EuroPrevall study has been described in detail previously [25, 26]. In summary, children were included around birth and standardized measurements were performed by questionnaires until 2.5 years of age. All children with symptoms suggestive of CMA underwent, among others, serum specific IgE (sIgE) measurement (Phadia Diagnostics, Uppsala, Sweden) and a double blind placebo controlled food challenge (DBPCFC). sIgE-values <0.35 kU/L, were considered negative. Healthy control children were selected from the entire Dutch cohort. Controls had no symptoms suggestive of any food allergy nor suffered from atopic dermatitis. In children with CMA and healthy controls, an attempt was made to obtain one full blood sample (EDTA KE 2.6 mL Monovette, Sarstedt BV, Etten-Leur, Netherlands) for DNA-isolation, besides the blood used for sIgE measurement. The Medical Ethics Committee of the Academic Medical Hospital (METC 06/005) approved the Dutch EuroPrevall Birth Cohort Study, including (epi) genetic studies. Written informed consent, both for the study and for the epigenetic study, were obtained from both parents of each child, unless only one of them had parental rights.

Patient characteristics were analysed with *t* test (Mann–Whitney U test when data was not normally distributed) for continuous parameters. Chi square test was used for categorical variables and Chi square test for trend for multiple categories categorical variables. P values <0.05 were considered significant. SPSS version 20 was used (IBM SPSS Statistics for Windows, Armonk, NY).

### CMA diagnosis

DBPCFC is the gold standard for diagnosing CMA and the challenge of choice according to the study protocol [25–27]. The DBPCFC has been described in detail previously [28]. In summary, the child receives cow’s milk protein (CMP) free formula with added CMP (active day) or the same CMP free formula without the added CMP (placebo day) in increasing doses, double blinded, in a random order, on two different days. All children suspected of CMA were challenged with a DBPCFC. DBPCFC was repeated annually until the child was tolerant for CMP [25]. Meaning, the DBPCFC was negative and/or the child was consuming CMP regularly without symptoms.

### Samples selected for epigenetic analysis

**Fig. 1:**
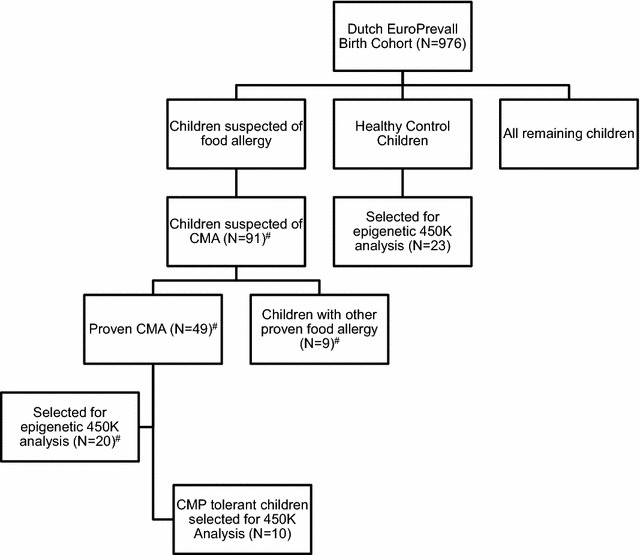
Flowchart of samples selected for our epigenetic and phenotypic studies. *CMA* cow’s milk allergy, *N* number, ^#^selected for phenotypic factor analysis

DNA samples of 20 children with proven and active CMA at the time of blood drawing were selected. Samples of healthy controls (N = 20) were gender matched with the 20 CMA-samples. Also ten DNA samples were selected of “tolerant boys”. These boys had proven CMA, but who were already CMP-tolerant at DNA-sampling. For the tolerant-children only boys were included, due to the limited availability of samples of tolerant girls. Samples of these children have also been studied in a different study on genetic aberrations regarding the Filaggrin-gene and SNPs known in allergic diseases [29]. A flowchart of the selected samples can be found in Fig. 1. To our knowledge no epigenome wide specific power calculator is available. Although not fully appropriate, we estimated the statistical power of our sample using a power calculator webtool, originally designed for expression array experiments (http://bioinformatics.mdanderson.org/MicroarraySampleSize). The following thresholds were used: Number of genes (25,000); Acceptable number of false positives (1); desired fold change (1.3); desired power (0.8); standard deviation (0.2). Using these parameters a sample size of 14 would be sufficient to detect an alpha of 0.00004 per gene.

### Epigenetic analysis of selected samples

DNA was isolated from whole blood. Bisulfite treatment (ZYMO^®^) was applied on genomic DNA. DNA-methylation profiles for each sample were obtained using the 450K infinium array of Illumina. Raw data was processed using standard “genome studio” software of Illumina. This processed data was analyzed with statistical analysis scripted in “R” and “Perl”. “R” packages IMA, Minfi, wateRmelon, lumi and globaltest were used. We corrected for the two types of probes present on the Illumina array using BMIQ normalization. The presence of technical and/or biological batch effects was estimated and evaluated by means of principal component analysis (PCA). QQ-plots were generated, deflation or inflation factor ranging between 0.97 and 1.3 was assumed to be acceptable. Relative blood cell distribution was evaluated using the Minfi package according the method of Houseman et al. [30]. Standard output of the relative blood cell distribution comprises only CD8+ and CD4+ T cells, Natural killer cells, B cells, Monocytes and Granulocytes. Since CMA is an immunological disorder, blood cell distribution is related to our factor of interest. Therefore adjustment for blood cell distribution was assumed inappropriate. Statistical analysis on individual probe differentially methylated positions (DMPs) and on specific annotated differentially methylated regions (DMR) within a gene was performed using IMA package. For association analysis a linear model (limma; default settings) was used. All models were adjusted for age and where necessary the model was adjusted for gender. In the combined analysis probes representing chromosome X and Y were removed from the dataset. Analyses were conducted for children with CMA versus control children (combined analysis), girls with CMA versus control girls (girls), boys with CMA versus control boys (boys), and tolerant boys versus control boys (tolerant). In order to explore our CMA datasets we evaluated general methylation differences. For exclusion of the majority of non-informative probes we used solely probes with an unadjusted p < 0.05 and an absolute methylation difference >5 % in these analyses. Recent published information on the presence of common single nucleotide polymorphism (SNPs) beneath the Illumina 450K DNA-methylation probe and specificity of the probe was post hoc added to the output using in-house developed “Perl” scripts [31]. Common SNPs are especially effective confounding in DMPs. For the hypothesis free analysis approach we evaluated DMPs and DMRs, for the hypothesis driven analysis approach we extracted all individual probes within the candidate gene. Selection of candidate genes is described below. Post-hoc SNP evaluation was preferred over prior excluding common SNPs from the analysis dataset. DMPs were evaluated according the adjusted p values (Benjamini–Hochberg), an adjusted p value of <0.05 was assumed significant. The annotation of methylation regions used for DMR analysis in the IMA package is predefined and thus not based on biological/statistical correlation between CpGs. Moreover, IMA’s adjusted p values are calculated per region. Unfortunately, many of those regions overlap, which results seemingly in more independent tests and thus a false inflation of the multiple test penalty. In other words, we hypothesized that such annotation definition implicated more false negative findings per definition. Therefore our multiple test correction was based on a Bonferonni correction using p value of 0.05 divided by the maximum amount of genes (i.e. maximum number of genes found was 19,500) found in association analyses of this study. As such, DMRs were considered significant with p < 2.6 × 10^−6^.

Pathway analysis was performed in the combined, girls and boys groups using a global, untargeted, test method and the “Gene Ontology” (GO) databases [32]. Probes were filtered on the presence of entrez ID annotation. Entrez ID covered by less than 3 probes were excluded from the analysis. In our enrichment studies we used an arbitrary threshold of p < 0.01 in order to evaluate only the upper top and unique gene sets. Although enrichment or pathways that are plausible connected to the phenotype of interest is favorable, conclusions drawn from such analysis have to be taken with much greater caution compared to conclusions dawned from results like DMP and DMR analyses.

Illumina 450K DNA-methylation array profiles of all samples are available on request.

### Epigenetic validation procedure

Promising aberrant methylated regions were verified using Roche ^®^ 454 Next Generation Sequencing technology. The selection of these regions was based on the epigenome wide association results (top hits per group), feasible biological relevancy to CMA of the candidate and availability of DNA. Regions in the following genes were evaluated using multiple bisulphite primers sets: *DHX58*, *ZNF281*, *EIF42A* and *HTRA2*. Bisulphite primers were designed using Methprimer [33]. Primer information is described in Additional file 1: Table S1a. Cases and controls were random divided over 15 ID sequences, i.e. mids (Additional file 1: Table S1b). Concentration of polymerase chain reactions (PCR) fragments was obtained using “Lab on a Chip”. Subsequently PCR products were normalized and pooled. Sequence reactions were performed conform the Roche 454 protocol. Raw sequence data was mapped and aligned using the AvA software of Roche (Roche, Branford, United States). In-house developed scripts (*PERL*) were used to obtain methylation index (β) values for each CG position. QC of Roche 454 data was implemented in the script and was based on the presence of heterogeneous (non CG or TG) polymorphic sites (50 % distribution). Since bisulfite pyro sequencing is prone to a T-stretch bias, which may result in position shifts of the CG site, careful evaluation of flanking positions in the Roche 454 data was implemented in the script. Statistical analysis of the methylation index was performed in SPSS v20 using a Students T test. Pearsons correlation was used to evaluated the CG set within every amplicon. P values of correlated CG sites were combined using Fishers method combined p value method, p values <0.05 were assumed significant.

### Phenotypic differences between several subgroups of allergic children

To detect phenotypic differences between genders: factor analysis was performed. Out of the group of children suspected of CMA (N = 91), there were 49 children with CMA and 33 children without food allergies. The 20 children with CMA who were selected for epigenetic analysis, were part of the group of 49 children with proven CMA (Fig. 1). Phenotypic counts on skin, gastrointestinal, and respiratory symptoms and number of allergies in all subsets of patients were investigated. Symptoms included in each category have been described previously [28]. Number of allergy was defined as more than 1 challenge proven food allergy.

Factor analysis for phenotypic differences in all subsets of patients were performed by univariate analysis. Factor regression coefficients and gender were dependent variables and age was a covariate. P values <0.05 were considered significant. SPSS version 20 was used (IBM SPSS Statistics for Windows, Armonk, NY).

### Hypothesis driven (candidate gene) epigenetic association

Candidate genes were selected from a recent review of Bønnelykke et al. on allergy, sensitization, and related atopic disorders [34]. This review discusses previously reported genetic associations; mainly GWAS reports, with atopic disorders and/or sensitization mechanisms of approximately the last decade. We selected in total 66 genes previously associated with atopic disorders from large genome wide studies (GWAS) and 38 genes were associated with monogenic allergy related syndromes of which 22 genes were located at 22q11.1. 8 selected genes were reported for monogenic allergy related syndromes as well as GWAS (Additional file 1: Table S2). Individual probes were evaluated according the p value (hypothesis free) and adjusted p values based on p value of 0.05 divided by the maximum amount of genes. Since replication reports from which we obtained candidates followed not the same design and/or platform, we decided to evaluate these three reports independently, to overcome a too strict multiple test penalty when combining all three sets of hits. Therefore we used three separate levels of significance. Berni Canani et al. reported about 4 genes, Martino et al. reported about 49 genes and the review of Bønnelykke et al. reported about 66 genes, yielding significance thresholds of p < 1.25 × 10^−2^, p < 1.02 × 10^−3^ and p < 7.58 × 10^−4^ respectively.

## Results

In Table 1 the patient characteristics of the children selected for epigenetic analyses are shown. Controls and children with CMA were well matched except for age at DNA-sampling, in which children with CMA were significantly younger (p = 0.008) compared to controls. Our CMA group consists of both IgE as well as non-IgE mediated CMA, with slightly more IgE positive children (specific IgE > 0.35 kU/L, p = 0.044) in the CMA group compared to controls. Comparison of the 10 tolerant boys with 10 control boys, shows no significant difference in age at DNA-sampling (p = 0.71) nor IgE status (p = 0.56), but did show a significant difference in maternal folic acid use during pregnancy (p = 0.012), with more folic acid use in the control group.

**Table 1:** Characteristics of Dutch allergy study population; cow’s milk allergy versus controls

	CMA	Controls	p	Tolerant	Tolerant control boys	p
N (♀)^a^	20 (8)	20 (10)	0.342	10 (0)	13 (0)	NA
Age diagnosis CMA ± SD^b^	6.5 ± 2.5	NA	NA	5.8 ± 2.2	NA	NA
Age at sampling ± SD^b^	11.8 ± 4.8	17.2 ± 7.1	*0.008*	18.0 ± 5.5	17.2 ± 4.3	0.714
N of children with CMP sIgE > 0.35 kU/L^c^	7	1	*0.044*	2	1	0.560
Mean CMP sIgE value ± SD (kU/L)	1.15 ± 3.6	0.16 ± 0.64	0.260	0.19 ± 0.39	0.23 ± 0.79	0.693
Range CMP sIgE value (kU/L)	16.4	2.9	NA	1.0	2.9	NA
Maternal smoking^d^			0.659			0.483
Pregnancy	2	3		1	2	
Before pregnancy	5	5		2	4	
Never	13	12		7	7	
Maternal vitamin D^d^			0.317			NA
Regular	0	0		0	0	
Period only	0	0		0	0	
Irregular	1	0		0	0	
None	19	20		10	13	
Maternal multivitamin^d^			0.836			0.883
Regular	12	11		1	5	
Period only	2	2		4	3	
Irregular	1	1		0	0	
None	5	6		5	5	
Maternal folic acid^d^			0.656			*0.012*
Regular	8	9		0	5	
Period only	9	9		10	6	
Irregular	1	0		0	0	
None	2	2		0	2	

Significant p-values <0.05 are in italic

*NA* not applicable, *CMA* cow’s milk allergy, *p* p value, *SD* standard deviation, *N* number, *CMP* cow’s milk protein

^a^Pearson Chi square test

^b^Age in months ANOVA

^c^Fisher’s exact test, 2-sided

^d^Pearson Chi square test for trend

### Epigenetic 450K analysis of selected samples

**Fig. 2:**
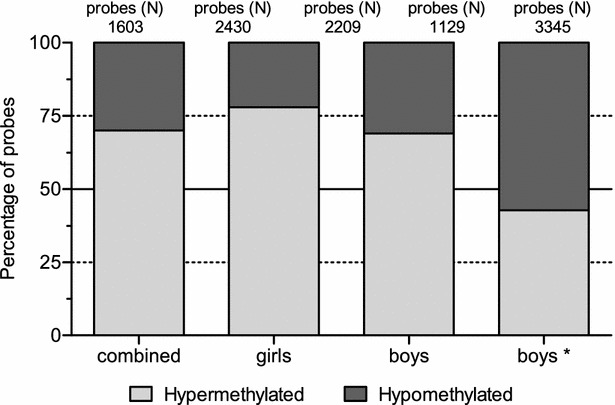
Percentage hyper and hypomethylated probes in cow’s milk allergy (CMA) patients versus controls. Filtering was based on a p value <0.05 and an absolute DNA-methylation difference (delta β values) >5 %; boy*: group boys who had CMA, but are now tolerant

All 450K analysed samples passed quality control. PCA showed no evidence of confounding technical batch effects according array or array-position (data not shown).General DNA-methylation analysis showed a hypermethylation in the CMA group. While in the tolerant boys versus controls we observed no deviation of the expected ratio hyper/hypomethylation (i.e. 50/50 %) (Fig. 2). A good concordance between CMA-children and controls with regard to the mean relative distribution of different types of, only the most abundant, blood cells: CD8+ and CD4+ T cells, natural Killer, B lymphocytes, monocytes and granulocytes was found (Additional file 2: Figure S1). Furthermore, the variation among blood cell type between groups (expressed as standard deviation) was not different. Relative cell distribution was not included as covariate in further analysis.

Top 5 hits for DMP’s between CMA and controls for all analysed groups are presented in Table 2. None of individual probes reached genome wide significance. Table 3 shows DMR’s that were found to be associated in more than one group (combined, girls and boys) with a delta methylation difference >0.01. In total ten regions, with their respective gene, were found. Additional file 1: Table S3a–c describes all DMR top 5 hits in each analysed group regardless of the methylation difference (delta).

**Table 2:** Top 5 differential methylated positions (DMPs) in combined, girls and boys analysis

Probe	p	Adj. p	Delta	Gene	Region
Combined					
cg07687119	6.4E−06	0.942	0.009	HOXC4/5/6	5UTR/body/body
cg01599189	1.1E−05	0.942	0.020	NBPF1	5′UTR
cg09789536	2.8E−05	0.942	0.020	KLHL17	Body
cg11433629	2.9E−05	0.942	−0.012	*IGR*	NA
cg23339709	2.9E−05	0.942	0.009	OR8U8/OR5M8	Body/exon1
Girls					
cg23876832^a^	1.7E−07	0.081	0.214	*IGR*	NA
cg13181291	1.8E−06	0.427	−0.016	GABPB2	Body
cg13700939	1.3E−05	0.999	0.065	*IGR*	NA
cg09573389	1.3E−05	0.999	0.039	C10orf58	TSS1500
cg24691332	3.0E−05	0.999	0.024	PARD3	Body
Boys					
cg06644124	1.6E−06	0.773	0.034	ZNF281	1stExon/5UTR
cg11126313	1.2E−05	0.997	0.009	CCR3	5UTR
cg09357684	1.4E−05	0.997	0.064	THAP3	TSS1500
cg09175834	1.5E−05	0.997	0.018	NAV3	1stExon
cg21255128	2.1E−05	0.997	0.018	C7orf50	Body

*p* p value, *adj. p* adjusted p value (BH), *Delta* expressed as β difference, *IGR* inter genic region, *5′*-*UTR* 5′ untranslated region, *Body* genebody, *TSS1500* transcription start site 1500 bp upstream

^a^High frequency (CEU MAF > 0.05) SNP located at position 34 of probe. Adjusted p values <0.05 were assumed significant

**Table 3:** Selection of most significant differential methylated regions found in >1 group with delta >0.01

Gene^a^	Chromosome	Delta	p	Overlap	Function obtained from genecards	Val^b^
OR5M8	11	0.010 0.012	7.7E−06 1.6E−04	Exon1 (combined) Exon1 (girl)	Codes for olfactory receptor located in the nose. Is involved in many neurotransmitter and hormone receptors and are responsible for the recognition and transduction of odorant signals	No
ZNF281	1	0.024 0.034	1.2E−04 1.3E−06	Exon1 (combined) Exon1 (boy)	Transcription repressor. Represses the transcription of a number of genes including GAST, ODC1 and VIM	Yes
KIAA1324L	7	0.012 0.016	4.8E−04 3.2E−04	Exon1 (combined) Exon1 (boy)	Estrogen-Induced protein-coding gene	No
EIF4E2	2	0.027 0.036	2.6E−05 1.0E−04	Gene body (combined) Gene body (girl)	Diseases associated with EIF4E2 include lung cancer, and among its related super-pathways are Interferon Signaling and Transcription Receptor-mediated HIF regulation	Yes
HTR2A	13	0.051 0.069	6.4E−05 8.1E−05	Gene body (combined) Gene body (girl)	Receptors for serotonin, a hormone that functions as a neurotransmitter and a mitogen. This receptor is involved in tracheal smooth muscle contraction, bronchoconstriction, and control of aldosterone production	Yes
ZNF366	5	0.022 0.033	2.2E−05 1.6E−04	TSS1500 (combined) TSS1500 (girl)	Transcription repressor. Associated diseases include breast cancer, and prostatitis	No
DHX58	17	−0.061 −0.061 −0.085 −0.085	3.1E−04 3.1E−04 3.3E−04 3.4E−04	3′-UTR (combined) SSHELF (combined) 3′-UTR (boy) SSHELF (boy)	Involved in the innate immune response to various RNA viruses and some DNA viruses	Yes
PRB4	12	−0.012 −0.015	6.3E−04 5.2E−04	3′-UTR (combined) 3′-UTR (boy)	The protein encoded by this gene is a proline-rich salivary protein	No
DNM1	9 130996210–130996443	−0.022 −0.028	7.2E−05 2.4E−04	ISLAND (combined) ISLAND (boy)	The encoded protein possesses unique mechanochemical properties used to tubulate and sever membranes, and is involved in clathrin-mediated endocytosis and other vesicular trafficking processes	No
WNT10A	2 219762987–219763537	0.038 0.039	9.0E−05 4.1E−04	SSHELF (combined) SSHELF (boy)	These proteins have been implicated in oncogenesis and in several developmental processes, including regulation of cell fate and patterning during embryogenesis	No

^a^Nearest gene; for genomic regions concerning ISLAND, NSHORE, SSHORE, NSHELF and SSHELF, UCSC browser was used to determine the nearest gene

^b^ *Val* validated using Roche 454 sequencing

We observed no overlap in loci between boys and girls. The locus *ZNF281* was found to be hypermethylated significantly in the boys analysis (p = 1.3 × 10^−6^, delta = 0.034). The remaining 9 loci showed nominal significant methylation differences and included *OR5M8*, *KIAA1324L*, *EIF4E2*, *HTR2A*, *ZNF366*, *DHX58*, *PRB4*, *DNM1* and *WNT10A* (Table 3). Although these loci were good candidates for validation procedures, the availability of sufficient DNA was a strong limiting factor. Therefore only four candidate loci were selected for validation. The following four genes were selected for validation: (1) *ZNF281*; involved in the transcriptional regulation of several interesting genes like *GAST*, (2) *EIF4E2*; involved in the interferon pathway, (3) *HTR2A*; involved in smooth muscle contraction and aldosterone production and (4) *DHX58*; involved in the innate immune response to RNA and DNA (viruses).

### Pathway evaluation of 450K data of selected samples

**Fig. 3:**
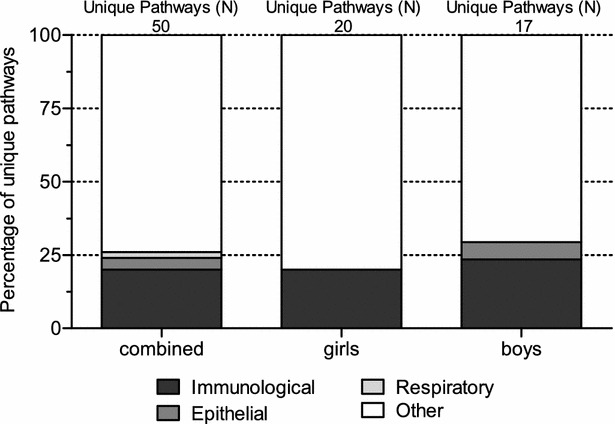
Global test and gene set enrichment analysis in CMA patients versus controls according GO terms. *Y*-*axis* contribution (%) of CMA related pathways, i.e. immunological, respiratory and epithelium related

Biological Process pathways, i.e. GO terms, with a nominal p value <0.01 were evaluated in the combined, girls and boys analysis according to a global, untargeted, test method. Entrez IDs covered by less than 3 probes were excluded from the analysis. We selected enrichment of GO terms representing a unique set of genes in combination with the number of contributing probe sets within that GO annotation. This way similar defined pathways but with different GO annotation were removed from the datasets. We hypothesized that GO terms involved in immunological, epithelial and respiratory classes are feasible to be involved in CMA. Therefore, we applied such class annotation to the detected GO terms. Figure 3 illustrates the relative percentage of the three classes within the filtered global test analysis in the combined, girls and boys groups. Specific GO term annotations are described in Additional file 1: Table S4.

The majority of detected pathways in the combined group involved immunological pathways (20 %), epithelial (4 %) and respiratory (2 %) pathways. In girls, only immunological pathways were detected (20 %). Boys on the other hand, showed in addition to immunological GO terms (23.5 %), epithelial related GO terms (5.9 %) as well. Interestingly, GO terms showed virtual no overlap between combined and girls or between girls and boys, while immunological terms between combined and boys suggests overlap in GO terms involved in epithelial cell polarity and immunological pathways.

### Validation of 450K results

Validation for the following candidate regions was performed: *ZNF281*, *EIF4E2*, *HTR2A and DHX58*. Association values of the DMRs of these four genes are described in Table 3. Bisulphite primer sets were designed overlapping or near the CpG extension site of (nominal) significant found 450K probes. In total we designed 12 different amplicons for the four loci. For *ZNF281* we were able to successfully optimize two amplicons, for *EIF4E2* and *HTR2A* one amplicon and for *DHX58* three amplicons. The exact chromosomal position (HG19) of covered CpGs as well as successful primer sequences are described in Additional file 1: Table S1a. PCR optimization was performed using a standard annealing temperature gradient protocol. 454 Sequencing preparation of the library was conform the Roche manual. Indices are presented in Additional file 1: Table S1b. The 450K DNA-methylation array data showed for several candidates methylation levels close to 10 or 90 %. The latter implies a lower reliability of the quantitative level of DNA methylation in case of a next generation sequencing (NGS) read count below 500. Therefore we aimed for an average depth of 1000 reads. Amplicon 2 and 3 of the *ZNF281* gene covered the 450K cg26678649 and cg09255666 probes and the flanking cg24347950 and cg0644124 probes. Unfortunately the average methylation index of the amplicon CpGs did not correspond with the 450K array data, indicating a failed validation for the 450K array *ZNF281* region data (Additional file 3: Figure S2A). The second amplicon for the *EIF4E2* region flanked the 450K cg10364945 probe. Although the methylation pattern of the CpGs present in this amplicon were concordant to the 450K data we did not observe a similar methylation variation between girl CMA cases and girl controls (Additional file 3: Figure S2B). For the *HTR2A* region one amplicon was successfully optimized, covering in total 6 CpG positions of which 5 covered the 450K probes cg20102280 (47470793), cg15894389 (47470857), cg02250787 (47470989),cg06476131 (47471052) and cg06476131 (47471090). The first CpG sites located at position 47470760, 47470793, 47470857 showed high correlation and showed a significant hypermethylation (p = 5.1E−4) in CMA patients (Additional file 3: Figure S2C). The first DHX58 amplicon, showed a concordant DNA-methylation pattern with the upstream flanking the probe cg08577293 and the downstream flanking probe cg01405890 covered in total 4 CpG sites. In line with the 450K data, the NGS data showed significant hypomethylation in CMA boys compared to controls (p = 1.1E−3). In CMA girls hypomethylation at this position was absent. The second DHX58 amplicon covered in total 22 CpG sites covering *and* flanking the 450K probe cg02450064 (40260053). Although, the NGS obtained methylation index of this CpG site showed moderate replication with regard to the 450K data, an interesting and highly significant hypomethylation was observed upstream of cg02450064 (40260053) in CMA boys (p = 9.8E−06) and CMA girls (p = 2.8E−06) compared to controls (Additional file 3: Figure S2D). The third *DHX58* amplicon showed insignificant but concordant methylation indices (0–5 %) compared to the 450K data (Additional file 3: Figure S2D).

### Gender differences

Epigenetic results show clear differences between CMA boys and girls with respect to their controls with hardly any overlap in obtained loci between the groups, as shown in Tables 2, 3, Fig. 3, and Additional file 1: Table S3. Although such gender difference, even with exclusion of the X and Y-chromosomes is a common phenomenon in epigenetics, we evaluated whether such difference is also phenotypically present. A factor-analysis on phenotypic CMA characteristics of all 91 children suspected of CMA, showed a difference in the univariate analysis with gender as independent factor (p = 0.05). The same factor-analysis model in the children selected for epigenetic analyses showed no difference in the univariate analysis with gender as independent factor (Tables 4, 5). Furthermore, there were more boys compared to girls with proven CMA (Table 4).

**Table 4:** Characteristics Subsets Dutch allergy study population

Subset	N (%♀)	Mean age ± SD (months)	Phenotype counts (%)			Allergies (>1)
			Skin	GI	Resp	
Suspicion of CMA allergy	91 (37.4)	36.2 ± 10.6	68 (55.7)	47 (38.5)	7 (5.7)	14
CMA	49 (36.7)	28.9 ± 13.7	35 (53.0)	27 (40.9)	4 (6.1)	7
CMA-subset	20 (40.0)	28.6 ± 11.4	15 (51.7)	11 (37.9)	3 (10.3)	7
FA	58 (34.5)	30.7 ± 14.2	44 (56.4)	29 (37.2)	5 (6.4)	14
No FA	33 (42.4)	45.9 ± 26.2	24 (54.5)	18 (40.9)	2 (4.5)	0

*SD* standard deviation, *FA* food allergy, *CMA* cow’s milk allergy, *CMA*-*subset* 20 children selected for epigenetic analysis from the total group of children with proven CMA, *GI* gastrointestinal, *Resp* respiratory

**Table 5:** Association of gender with factor analysis regression coefficients

Subset components^a^	Skin	GI	Resp	FA^b^	p value^c^
Allergy (C1 of 2)	0.85	−0.81	0.37	−0.02	0.051
Allergy (C2 of 2)	0.07	−0.18	−0.52	0.85	0.702
No FA (C1 of 2)	−0.73	0.85	0.20	0.49	0.794
No FA (C2 of 2)	0.51	0.15	0.91	0.12	0.292
FA (C1 of 2)	0.81	−0.82	0.47	0.26	0.284
FA (C2 of 2)	0.12	−0.02	−0.54	0.85	0.249
CMA (C1 of 2)	0.81	−0.81	0.52	−0.20	0.324
CMA (C2 of 2)	0.22	−0.20	−0.29	0.91	0.107
CMA-DNA (C1 of 2)	0.83	−0.86	0.30	0.51	0.843
CMA-DNA (C2 of 2)	0.16	0.02	0.85	−0.72	0.213

*FA* food allergy, *CMA* cow’s milk allergy, *GI* gastrointestinal, *Resp* respiratory

^a^Factor analysis, extraction of components: eigenvalue >1

^b^Univariate model: independent: gender, dependent: Regression coefficient factor analysis, covariate: age

^c^Reported p value for gender

### Hypothesis driven epigenetic association

For the candidate gene association studies we evaluated findings of three recent published reports with our dataset. First was the report of Berni Canani et al. on DNA methylation differences of *IL*-*4*, *IL*-*5*, *IL*-*10*, and *INF*-*γ* in IgE mediated CMA patients [35]. We did not detect any replication in any of the reported loci (data not shown) applying a multiple test significance level of p < 1.25 × 10^−2^ (Bonferonni, 0.05/4 independent tests). Second was the report of Martino et al. epigenome wide association in IgE mediated food allergy [36]. In total 2281 probes were extracted from our dataset. The six lowest p values (p < 0.005) covered 1 probe at *GALNTL4*, 2 probes at *HDAC4* locus, 1 probe at *KCNN3* locus and 2 probes at the *RPS6KA2* locus (data not shown). However, we cannot claim replication for any of the probes annotated for the 49 reported genes (p < 1.02 × 10^−3^, Bonferonni, 0.05/49 independent tests). The final hypothesis driven evaluation was based on a review on genetic rather than epigenetic association studies of atopic diseases by Bønnelykke et al. [34]. In total we selected 66 genes covered by approximately 1650 individual probes (Additional file 1: Table S2a). We found one significant replication applying a multiple test significance level of p < 7.58 × 10^−4^ (Bonferonni, 0.05/66 independent tests). The probe cg03990811, located in the *MICA* gene showed significant association (p = 3.9 × 10^−4^) in the combined analysis (Additional file 1: Table S4b). This finding is strengthened by the fact that we found a second probe (cg14462939, p = 3.9 × 10^−3^) present in the top5 hits and located in the *MICA* gene (Additional file 1: Table S2b).

## Discussion

In this pilot study we aimed to detect DNA-methylation differences between a group of mixed gender CMA and healthy controls. Several differential methylated loci were found, suggesting aberrant DNA-methylation is involved in the expression of CMA. Additional DNA-methylation analysis between a group of CMA tolerant boys and controls showed no DNA-methylation differences at all. We validate three loci of the top 10 of the 450K array results using bisulphite amplicon NGS. We found no evidence of confounding effects of inoculation or differences of blood cell distribution between cases and controls. Epigenetic gender differences were not reflected in a phenotypic symptom pattern in the CMA group but were present in the better-powered total allergy cohort.

### Epigenetic results

Although it is a topic of high interest, we are the second to describe an epigenetic survey in children with CMA [14, 35]. General hypermethylation was found in children with CMA, which disappeared after developing tolerance. In addition a difference in methylation between boys with CMA and control boys was found in this study. Hypermethylation in general is associated with repressed gene transcription [37]. In case a repressed gene codes for a transcription factor, several of its target genes might show an excess of expression. In CMA the immune system is overactive, suggesting immunological suppressive genes should be hypermethylated in general, compared to non-CMA children [38]. In CMA tolerant children this hypermethylation effect completely disappeared. Therefore, hypermethylation can only serve as biomarker during the allergic state.

DMR analysis showed several feasible genes to be involved in CMA. ZNF281 was significantly hypermethylated in boys versus controls. This gene is a transcriptional regulator, known to repress several genes, for example *GAST* (located on chromosome 17) [39]. *GAST* is known to stimulate the stomach mucosa, production of digestive enzymes, smooth muscle contraction and releases histamines. Therefore hypermethylation of *ZNF281* might be related to the gastro-intestinal symptoms in children with CMA. However, since the NGS amplicon analysis showed disconcordant and different methylation levels in CMA patients and controls, the claim of significance of the 450K results for the *ZNF281* locus has to be taken with caution.

*EIF4E2* is primarily involved in the initiation of protein synthesis [39]. Melnik et al. reported about the importance of breastmilk in early development and the mTORC1 signaling pathway. The EIF4 protein family plays a key role in this pathway that stimulates (post natal) growth and can be regulated by maternal excreted exosomal miRNAs [40]. Our finding of hypomethylation of *EIF4E2* might be the result of absence of these maternal miRNAs due to CMP free diet. Dysregulation of the mTORC1 pathway might be related to the methylation status of *EIF4E2*.

Hypermethylation of *HTR2A* is likely related to less expressed protein and might on turn be related to gastro-intestinal symptoms in CMA patients via altered intestinal muscle contraction [39]. The methylation pattern of the bsr amplicon NGS was similar to the 450K although we observed a consistent lower methylation index in the NGS data.

Although, not significantly associated according to the applied multiple test threshold, *DHX58* showed robust variability in all analyses including NGS bsr amplicon validation. The protein encoded by this gene plays a crucial step at the beginning of the cascade of antigen recognition in the innate immune response to various RNA viruses and some DNA viruses. Hypomethylation of *DHX58* might lead to over expression of this gene, which on turn might favour viral nucleoprotein recognition by DDX58/RIG-I and IFIH1/MDA5 complexes [39]. Deretic et al. proposed that nutritional signals (mTORC pathway) and immune signalling (RIG-I/MDA5 pathway) might stimulate similar cascades. This suggestion might indicated that our findings on *EIF4E2* and *DHX58* underlie similar and maybe shared cascades [41]. Future studies should therefore investigate whether cow’s milk proteins are capable of mimicking epitopes, confirming a role of *DHX58* in CMA.

Other genes in the top 5 findings are interesting as well. Unfortunately, the availability of DNA in these CMA samples was limited which disabled validation of further loci. Feasible biological relevance and their possible involvement in CMA have to be studied further.

### Pathway evaluation of 450K data of selected samples

Gene set enrichment analyses based on a global test method, suggest that specific immunological mechanisms are involved in CMA. Interestingly, no overlap of these pathways between combined and girls or girls and boys were observed, suggesting involvement of different immunological biological processes between boys and girls with CMA (Additional file 1: Table S4a; Fig. 3). However, limited statistical power cannot be excluded as cause of this difference between genders. We did observe gender differences with regard to other, non-immunological, biological processes. A limited number of biological processes suggests involvement of epithelium development in combined CMA and boys CMA only. Furthermore, biological processes involved in the respiratory mechanism, was solely observed in boys. We can state that our results strongly suggest involvement of gender specific immunological processes and that other processes prone to be involved in CMA, are likely to be different between genders as well.

### Gender difference

In this study we observed a clear difference, or in other words no overlap, of aberrant methylation loci, between boys and girls. These results were found in the differences in top-hits in the epigenetic results, but also in the number of boys with CMA and in the factor-analysis of all children suspected of CMA. This implies that different biological mechanisms underlie CMA in boys and girls. However we were not able to confirm this difference in the factor-analysis in our subgroup of 20 CMA-children and 20 controls. This is probably due to very small subgroups in a very heterogeneous disease. Furthermore symptom descriptions, obtained through questionnaires answered by parents are very subjective, compared to epigenetic results. It is very likely that these effects are smaller in larger sample sizes. However, differences between boys and girls are not unexpected. It has been described both, in allergy studies, as well as in epigenetic studies [21–24].

### Allergic march

The epigenetic results presented in this study, show methylation differences in children suffering from CMA relative to controls, which disappears in the children who developed tolerance to CMA.

So far, it has been considered that children who suffer from FA, are at risk of developing other allergic diseases later in life [42]. However, since the methylation differences found in our study disappear upon developing CMP tolerance, there is no indication that the studied loci are involved in development of the allergic march in children with CMA, despite some (top hit) loci have been described in childhood asthma as well [39]. A recent review discussed the short-term dynamics of epigenetic programming within human immunological responses. They stated that epigenetic regulation plays a key role in proliferation and differentiation of specific immune systems. Immune cells share for example many specific transcription factors, but remodelling of the epigenome is thought to be essential regulating specific gene expression profiles for different lineages [43]. Since our data suggest the absence of CMA “epigenetic memory” or evidence of certain enrichment of specific immune cells, it may disprove the hypothesis arguing for an epigenetic basis underlying the “allergic march”. Literature on the development of the allergic march is contradicting, with studies reporting on the development of the allergic march, as well as studies describing a co-manifestation instead of an allergic march [9, 42, 44, 45]. An epigenetic risk profile might also be a contributing factor. Since methylation can change back and forth more than once in life, certain people might be more at risk for expressing diseases involved by epigenetic changes. However, our results need to be interpreted with great caution, with a very little sample size in a heterogeneous disease. Furthermore, these results are based on CMA and CMP-tolerance development only, clinical data regarding development of other allergic diseases in life are not yet available, due to the prospective character of this study. It would be interesting to evaluate these children in the future in case of the development of other allergic diseases.

### Replication other reports involving CMA

Although no significant replication was found with the review of Bønnelykke et al., according the q value based on the number of selected probes, applying a multiple test correction based on the number of genes, showed significant replication for the *MICA* gene [34]. In addition, this finding is strengthened by the fact that another probe located in this gene was present in the DMP top 5 hits. According UCSC public databases, both probes are located in the first intron of the *MICA* gene which, also represents a strong H3K27AC mark that is associated with an active regulatory element [46]. *MICA* is closely positioned to HLA-B and C on chromosome 6, which both are thought to play an important role in MHC interactions and immune related disorders. *MICA* itself codes for a protein that functions as a stress-induced antigen. Interestingly, *MICA* is broadly recognized by intestinal epithelial gamma delta T cells, favouring an association with FA.

We were not able to replicate the results of two other studies on epigenetics in food allergy studies [34, 35]. Differences in the design between our study and the studies of others are likely to be responsible for the absence of replication. First of all, we used whole blood sample analyses, while both other studies used subsets of blood cells, namely peripheral blood mononuclear cells and CD4+ T cell respectively. Second, we studied children with IgE and non-IgE mediated CMA, while Berni Canani et al. studied children with CMA with high levels of IgE only and Martino et al. studied children with IgE-mediated FA, and included only one child with CMA. Furthermore the study of Berni Canani et al. used a candidate gene approach. The study of Martino et al. was based on a pooled sample strategy and analysis was based on a mixed gender design [34, 35]. Our analysis was untargeted and we have followed both a combined and gender stratified strategy. This emphasizes the importance and the great bias potential of epigenetic, tissue related, heterogeneity which is very likely to block positive replication between studies.

### Strength and weaknesses of the study

The main strength of the studied samples is that CMA is diagnosed according to the current available gold standard [27]. Furthermore, clinical data are well documented due to the setup of the study with regular questionnaires [25].

Since our sample size is small, we used strict thresholds for evaluation of our results. For p values this threshold was based on bonferonni and for effect size we applied an absolute difference of >5 %. Despite these thresholds results need to be interpreted with great caution, until they are replicated in an independent study.

Our sample involves IgE and non-IgE mediated CMA cases. Additional analysis of epigenetic profiles of CMA patients with relative high levels of IgE (N = 12 combined genders, IgE ranging between 0.01 and 16.4, three CMA cases IgE > 0.5) showed no additional significant hits with regard to the total group of CMA children (data not shown). In general the IgE plasma level is not an essential parameter in CMA diagnosis. Moreover, IgE mediated and non-IgE mediated CMA might origin from an overlapping or similar sensitization mechanism [43]. Our study is thus limited in the sense that we solely are able to detect general CMA associated epigenetic loci. Future research using a larger CMA sample should include IgE and non-IgE stratified analyses. Ideally these IgE measurements should be measured several times during the course of disease.

Unfortunately, there was only one DNA-sample available per child. Therefore, comparisons over time within the same child were not possible. Also, it was not possible to obtain DNA-samples in all children. Furthermore the amount of blood drawing for research purposes in infants is in the Netherlands limited to 2.5 mL. The latter also implies that a sufficient DNA yield in DNA extraction of a specific blood cell is virtual impossible.

Proper meta-analysis nor replication of our findings in an independent well characterized CMA cohort unfortunately appeared not to be feasible. Previous reports generally are based on specific immune cells while our study is based on DNA obtained from whole blood. Validation our dataset was therefore solely based on replicating findings of others in our sample.

In general, discrepancy between the NGS and 450K methylation index might be the result of a failing normalization procedure (BMIQ) to overcome the probe bias of the array. A suboptimal primerset, in case of amplicon NGS, cannot be excluded as another cause of the discrepancy.

The national Dutch vaccination program is extensive and starts already at young age. Since vaccination can have a major impact on the blood cell distribution and/or on methylation status of particular loci we preselected our sample and evaluated post hoc the period between vaccination and blood drawing (Additional file 4: Figure S3). We excluded all children who received a vaccination up to 4 weeks prior to DNA-sampling [47]. Since no significant differences between CMA cases and controls and their period (>4 weeks) between vaccination and blood drawing were found, we are confident vaccination plays an insignificant role with regard to the epigenetic aberrations we detected in this study. Finally there is a statistical significant difference in the folic acid consumption of mothers during pregnancy between the tolerant boys and control boys. It is known that folic acid has the ability to influence epigenetics. Since our sample size is very limited, folic acid is widely used by pregnant women and this difference was only found between already tolerant boys and controls (and not in children still suffering from CMA), we consider the relevance of this difference limited [48–50].

### Future studies

The present study should be classified as pilot study or proof of principle study. Moreover, CMA can be characterized as a complex disease involving multiple loci and a heterogeneous phenotype. Thus, in combination with the fact we analysed only relative small groups, further studies in larger cohorts are essential in order to replicate or validate our findings. Ideally, sample size in this study would be large enough to investigate boys and girls as well as IgE and non-IgE mediated CMA separately or to perform subgroup-analysis.

To investigate whether there is a evidence for the allergic march, it would be interesting to compare epigenetic analyses during infancy, school-age and later life. Boys and girls should be separately analysed to also investigate a gender effect.

Since infants develop CMA at a very young age. Diet is known to be involved in the development of certain diseases, probably through epigenetic alterations, in the next generations [51]. And milk is known to influence the infants immune system [40, 52]. It would be very interesting to investigate the role of (breast)milk in a future epigenetic study in children with cow’s milk allergy, especially seen our findings in *EIF4E2*.

## Conclusion

Current studies indicate the involvement of epigenetic mechanisms underlying the expression of CMA. In particular DHX58, involved in DDX58/RIG-I and IFIH1/MDA5 immunological pathways, are involved in CMA in young boys, while in CMA girls *EIF4E2*, *HTR2A* seems to play a role. Our data strongly suggests a different mechanism for girls and boys underlying CMA. We have no indication DNA methylation aberrations play a crucial role in the allergic march, since all effects disappeared upon developing CMA tolerance in boys. However, due to small sample size further studies are necessary before definite conclusions can be drawn.

## Additional files

10.1186/s13601-016-0105-z

10.1186/s13601-016-0105-z Relative blood cell type distribution estimate in CMA patients and controls. CD8T: CD8+ T cells; CD4T: CD4+ T cells; NK: natural killer cells; Bcell: B cells; Mono: monocytes; Gran: granulocytes.
10.1186/s13601-016-0105-z Average methylation index. A: ZNF281 amplicon 2 and 3 in CMA boys and control boys. B: EIF4E2 amplicon in CMA girls and control girls. C: HTR2A amplicon in CMA girls and control girls. D: DHX58 amplicon 1–3 in CMA boys and control boys. Y-axis: Methylation index expressed in (normalized) β-values; X-axis: ranked chromosomal probe position according hg19.
10.1186/s13601-016-0105-z Categorical time span between vaccination and blood drawing (DNA) of CMA cases and controls.

## Abbreviations

CMA: cow’s milk allergy
CMP: cow’s milk protein
DBPCFC: double blind placebo controlled food challenge
DMP: differentially methylated positions
DMR: differential methylated regions
DNA: deoxyribonucleic acid
EWAS: epigenome-wide association studies
FA: food allergy
GO: gene ontology
GWAS: genome wide association studies
IgE: immunoglobuline E
NGS: next generation sequencing
PCA: principal component analysis
PCR: Polymerase chain reaction
RNA: ribonucleic acid
sIgE: serum specific IgE
SNP: single nucleotide polymorphism
