# Supplementary material for: Cow’s milk allergy in Dutch children: an epigenetic pilot survey
Source: Clin Transl Allergy. 2016 May 4;6:16. doi: 10.1186/s13601-016-0105-z (PMC4855719; doi:10.1186/s13601-016-0105-z)
Supplement: Supplementary file 1 — 10.1186/s13601-016-0105-z [file 13601_2016_105_MOESM1_ESM.doc]

**Table S1a: Primer and amplicon descriptions 454 ROCHE bisulfite sequencing**

| **Primer name** | **Sequence ^a^** | **Position ^b^** | **bp ^c^** | **CpG** |
| --- | --- | --- | --- | --- |
| ZNF281_2_F | *CGTATCGCCTCCCTCGCGCCATCAG***[MID]**TTTTTTTAGTTAGGTTTTTTT | Chr:1; 200378868 | 83 | 17 |
| ZNF281_2_R | *CTATGCGCCTTGCCAGCCCGCTCAG***[MID]**AATTCCATCTCTACCCTCCTACC | Chr:1; 200378785 |  |  |
| ZNF281_3_F | *CGTATCGCCTCCCTCGCGCCATCAG***[MID]**GTTTTTTTTGTTTTTGTTTTTT | Chr:1; 200379370 | 100 | 10 |
| ZNF281_3_R | *CTATGCGCCTTGCCAGCCCGCTCAG***[MID]**CAATATCTACTAAAAACTCCTC | Chr:1; 200379270 |  |  |
| EIF4E2_2_F | *CGTATCGCCTCCCTCGCGCCATCAG***[MID]**AGTGGAGTTGGTTTAAATTAGTTAAGTGT | Chr:2; 233432989 | 375 | 5 |
| EIF4E2_2_R | *CTATGCGCCTTGCCAGCCCGCTCAG***[MID]**AAAAAAATTAATAAAAAATACCTCTTTCC | Chr:2; 233433364 |  |  |
| HTR2A_2_F | *CGTATCGCCTCCCTCGCGCCATCAG***[MID]**TTTTAAATGTGTGTTTGTTGAGG | Chr:13; 47471097 | 331 | 6 |
| HTR2A_2_R | *CTATGCGCCTTGCCAGCCCGCTCAG***[MID]**ACAAACAACTTTCCTCCCTAAAA | Chr:13; 47470766 |  |  |
| DHX58_1_F | *CGTATCGCCTCCCTCGCGCCATCAG***[MID]**GGAGAGTTTGTAGTAGATTTTAGGTTTT | Chr:17; 40253765 | 331 | 4 |
| DHX58_1_R | *CTATGCGCCTTGCCAGCCCGCTCAG***[MID]**ATACCCAATTACACACTATACAAACAATTT | Chr:17; 40253434 |  |  |
| DHX58_2_F | *CGTATCGCCTCCCTCGCGCCATCAG***[MID]**GTTTATTTATTTTAGGGGTGGAGT | Chr:17; 40259949 | 355 | 21 |
| DHX58_2_R | *CTATGCGCCTTGCCAGCCCGCTCAG***[MID]**CCTATACCCTAACCTCTAACCTC | Chr:17; 40259594 |  |  |
| DHX58_3_F | *CGTATCGCCTCCCTCGCGCCATCAG***[MID]**TGGTTATAATTGATTGTAGAAGGGTTAG | Chr:17; 40264930 | 335 | 7 |
| DHX58_3_R | *CTATGCGCCTTGCCAGCCCGCTCAG***[MID]**CAAAACTCCACCCCACTTAAA | Chr:17; 40264595 |  |  |

^a^ Primer sequences were constructed according as follows: Roche reverse complement sequence primer, mid sequence (for sequences see suplemental table 3b) and amplicon specific primer.^b^ Positions (hg19) are annotated by the 5’start point. ^c^ Amplicon size including amplicon specific primers

**Table S1b: Primer seqeunces of 454 ROCHE mids.**

| **MID name** | **Sequence** | **MID name** | **Sequence** |
| --- | --- | --- | --- |
| **MID_1** | ACGAGTGCGT | **MID_9** | TAGTATCAGC |
| **MID_2** | ACGCTCGACA | **MID_10** | TCTCTATGCG |
| **MID_3** | AGACGCACTC | **MID_11** | TGATACGTCT |
| **MID_4** | AGCACTGTAG | **MID_12** | TACTGAGCTA |
| **MID_5** | ATCAGACACG | **MID_13** | CATAGTAGTG |
| **MID_6** | ATATCGCGAG | **MID_14** | CGAGAGATAC |
| **MID_7** | CGTGTCTCTA | **MID_15** | ATACGACGTA |
| **MID_8** | CTCGCGTGTC |  |  |

**Table S2a: 450K probe density candidate genes**

| Candidate gene* | Probes (N) i | Probes (N) ii | Probes (N) iii | Candidate gene* | Probes (N) i | Probes (N) ii | Probes (N) i |
| --- | --- | --- | --- | --- | --- | --- | --- |
| *ACTL9* | 12 | 12 | 12 | *NFATC2* | 37 | 37 | 37 |
| *ADA* | 8 | 8 | 9 | *NLRP10* | 6 | 7 | 6 |
| *ADAD1* | 19 | 20 | 19 | *ORMDL3* | 18 | 18 | 18 |
| *BCL6* | 54 | 55 | 54 | *OVOL1* | 28 | 29 | 28 |
| *C11orf30* | 20 | 20 | 21 | *PFDN4* | 13 | 14 | 13 |
| *CARD11* | 76 | 77 | 77 | *PGM3* | 20 | 20 | 21 |
| *CCDC80* | 19 | 19 | 20 | *PLCG2* | 33 | 35 | 33 |
| *CDK2* | 19 | 19 | 20 | *PLCL1* | 16 | 17 | 16 |
| *CLEC16A* | 67 | 68 | 67 | *PRR5L* | 44 | 44 | 44 |
| *DCLRE1C* | 19 | 19 | 20 | *PTGER4* | 23 | 23 | 24 |
| *DOCK8* | 7 | 7 | 8 | *PVT1* | 85 | 86 | 85 |
| *FLG* | 10 | 10 | 10 | *PYHIN1* | 9 | 10 | 9 |
| *FOXA1* | 19 | 19 | 19 | *RAG1* | 6 | 6 | 6 |
| *FOXP3* | 0 (chr. X) | 9 | 9 | *RAG2* | 14 | 14 | 14 |
| *GATA3* | 67 | 72 | 67 | *RANBP6* | 5 | 5 | 5 |
| *GLB1* | 22 | 22 | 22 | *RMRP* | 4 | 4 | 4 |
| *GSDMA* | 10 | 10 | 10 | *RORA* | 107 | 109 | 109 |
| *GSDMB* | 10 | 10 | 10 | *SLC25A46* | 16 | 16 | 17 |
| *HLA-A* | 39 | 41 | 39 | *SMAD3* | 57 | 58 | 57 |
| *HLA-B* | 46 | 47 | 50 | *STAT3* | 27 | 28 | 27 |
| *HLA-DQB1* | 50 | 48 | 52 | *STAT6* | 9 | 9 | 9 |
| *IKZF4* | 10 | 11 | 10 | *TLR1* | 5 | 5 | 5 |
| *IL13* | 9 | 9 | 9 | *TLR10* | 6 | 6 | 6 |
| *IL18R1* | 5 | 5 | 5 | *TLR6* | 11 | 11 | 11 |
| *IL1RL1* | 10 | 10 | 10 | *TSLP* | 18 | 18 | 18 |
| *IL2* | 2 | 2 | 2 | *TTC6* | 0 | 0 | 0 |
| *IL2RB* | 9 | 9 | 9 | *TXLNA* | 22 | 22 | 22 |
| *IL33* | 2 | 2 | 2 | *USP38* | 11 | 11 | 11 |
| *IL6R* | 25 | 27 | 25 | *WAS* | 0 (chr. X) | 9 | 9 |
| *LPP* | 97 | 98 | 97 | *ZBTB10* | 15 | 15 | 15 |
| *LRRC32* | 23 | 23 | 23 | *ZNF365* | 39 | 39 | 39 |
| *LSM3P4* | 0 | 0 | 0 | *ZNF652* | 15 | 16 | 15 |
| *MICA* | 79 | 80 | 80 |  |  |  |  |
| *MYC* | 34 | 36 | 35 | 66# | 1617 | 1665 | 1655 |

*Candidates selected from Bønnelykke *et al* (2015). #66 candidates were selected of which 64 were covered by the 450K array. (i) number of probes in combined analysis, (ii) number of probes in girls analysis and (ii) number of probes in boys analysis

**Table S2b: Candidate gene approach; top 5 probe hits in combined, gilrs and boys association analysis**

| **Combined** |  |  |  |  |
| --- | --- | --- | --- | --- |
| **Gene** | **Probe** | **P-value** | **q-value*** | **Bonferonni **** |
| MICA | cg03990811 | 3,9E-04 | 0,94 | **S** |
| ZNF365 | cg02712553 | 1,5E-03 | 0,94 | NS |
| MICA | cg14462939 | 3,7E-03 | 0,94 | NS |
| ZNF365 | cg14701867 | 3,9E-03 | 0,94 | NS |
| TLR10 | cg19398783 | 6,7E-03 | 0,94 | NS |
| **Girls** |  |  |  |  |
| **Gene** | **Probe** | **P-value** | **q-value*** |  |
| IL6R;IL6R | cg15633035 | 1,2E-03 | 0,99 | NS |
| SMAD3 | cg17184477 | 3,6E-03 | 0,99 | NS |
| PVT1 | cg02100150 | 5,2E-03 | 0,99 | NS |
| STAT3;STAT3;STAT3 | cg12873903 | 6,8E-03 | 0,99 | NS |
| PGM3 | cg07964958 | 7,5E-03 | 0,99 | NS |
| **Boys** |  |  |  |  |
| **Gene** | **Probe** | **P-value** | **q-value*** |  |
| MICA | cg03990811 | 1,3E-03 | 0,99 | NS |
| ZNF652;ZNF652 | cg20461826 | 2,9E-03 | 0,99 | NS |
| TXLNA | cg04031504 | 3,4E-03 | 0,99 | NS |
| ZNF365;ZNF365;ZNF365 | cg02712553 | 4,8E-03 | 0,99 | NS |
| CLEC16A | cg06906156 | 4,8E-03 | 0,99 | NS |

* q-value based on FDR probe set specific analysis

** Bonferonni multiple test correction threshold (0.05/64 genes = 7,8E-04)

**Table S3a**: 450K analysis of combined group; regional top 5 hits

| **Combined** | | | | | | | |
| --- | --- | --- | --- | --- | --- | --- | --- |
| **Nearest Gene** | **Chr** | **Region** | **Delta** | **P-value** | **Overlap** | **Delta (T)** | **P-value (T)** |
| **OR5M8** | **11** | **Exon1** | **0.010** | **7.7E-06** | **Exon1 (girl)** | NA | |
| **ZNF281** | **1** | **Exon1** | **0.024** | **1.2E-04** | **Exon1 (boy)** |
| **KIAA1324L** | **7** | **Exon1** | **0.012** | **4.8E-04** | **Exon1 (boy)** |
| NNMT | 11 | Exon1 | 0.013 | 6.2E-04 |  |
| ARHGAP23 | 17 | Exon1 | -0.015 | 6.4E-04 |  |
| HOXC6 | 12 | Gene body | 0.009 | 9.1E-06 | Gene body (boy) |
| **EIF4E2** | **2** | **Gene body** | **0.027** | **2.6E-05** | **Gene body (girl)** |
| NR1H3 | 11 | Gene body | 0.019 | 4.1E-05 |  |
| **HTR2A** | **13** | **Gene body** | **0.051** | **6.4E-05** | **Gene body (girl)** |
| JPH4 | 14 | Gene body | -0.023 | 4.4E-04 |  |
| ZNFX1 | 20 | TSS200 | 0.007 | 3.6E-04 |  |
| PDLIM7 | 5 | TSS200 | 0.005 | 5.1E-04 |  |
| ALAD | 9 | TSS200 | 0.010 | 6.8E-04 |  |
| CNRIP1 | 2 | TSS200 | 0.022 | 1.3E-03 |  |
| SCIN | 7 | TSS200 | 0.011 | 1.4E-03 |  |
| **ZNF366** | **5** | **TSS1500** | **0.022** | **2.2E-05** | **TSS1500 (girl)** |
| TWIST2 | 2 | TSS1500 | 0.009 | 6.0E-05 | TSS1500 (girl) |
| HSBP1 | 16 | TSS1500 | 0.033 | 1.6E-04 |  |
| GSS | 20 | TSS1500 | 0.027 | 1.6E-04 |  |
| TP53INP2 | 20 | TSS1500 | 0.007 | 3.6E-04 |  |
| **DHX58** | **17** | **3-’UTR** | **-0.061** | **3.1E-04** | **3-’UTR (boy)** |
| RNASEH2A | 19 | 3-’UTR | -0.013 | 3.4E-04 |  |
| LYZL4 | 3 | 3-’UTR | 0.021 | 3.5E-04 |  |
| ARL10 | 5 | 3-’UTR | 0.019 | 3.9E-04 |  |
| **PRB4** | **12** | **3-’UTR** | **-0.012** | **6.3E-04** | **3-’UTR (boy)** |
| **ZNF281** | **1** | **5-’UTR** | **0.012** | **4.0E-04** | **Exon1 (boy)** |
| ZZEF1 | 17 | 5-’UTR | 0.030 | 4.0E-04 |  |
| B4GALNT2 | 17 | 5-’UTR | 0.005 | 4.1E-04 |  |
| TATDN3 | 1 | 5-’UTR | 0.006 | 5.8E-04 | 5-’UTR (boy) |
| AES | 19 | 5-’UTR | 0.006 | 6.2E-04 |  |
| HOXC6 | chr12:54423427-54423712 | ISLAND | 0.009 | 4.3E-06 | Gene body (boy) |
| TWIST2 | chr2:239755096-239758310 | ISLAND | 0.009 | 4.7E-05 | TSS1500 (girl) |
| **DNM1** | **chr9:130996210-130996443** | **ISLAND** | **-0.022** | **7.2E-05** | **ISLAND (boy)** |
| EPN3 | chr17:48619111-48619794 | ISLAND | 0.006 | 2.7E-04 |  |
| GALNS | chr16:88905509-88905750 | ISLAND | 0.045 | 3.0E-04 |  |
| ADSL | chr14:55907197-55907433 | NSHELF | -0.034 | 3.3E-05 |  |
| C2ORF57 | chr17:48276877-48279008 | NSHELF | 0.027 | 9.3E-05 |  |
| EFR3B | chr19:1315775-1316092 | NSHELF | 0.008 | 1.3E-04 |  |
| CNNM2 | chr16:2255168-2255828 | NSHELF | 0.016 | 1.6E-04 |  |
| SLC25A38 | chr16:83841412-83841988 | NSHELF | 0.033 | 1.6E-04 |  |
| TBL2 | chr14:55907197-55907433 | NSHORE | -0.034 | 3.3E-05 |  |
| COL1A1 | chr17:48276877-48279008 | NSHORE | 0.027 | 9.3E-05 |  |
| EFNA2 | chr19:1315775-1316092 | NSHORE | 0.008 | 1.3E-04 |  |
| MLST8 | chr16:2255168-2255828 | NSHORE | 0.016 | 1.6E-04 |  |
| HSBP1 | chr16:83841412-83841988 | NSHORE | 0.033 | 1.6E-04 |  |
| BCLAF1 | chr6:136610148-136611285 | SSHELF | -0.012 | 2.2E-05 |  |
| LDLRAP1 | chr1:25869893-25871042 | SSHELF | -0.015 | 3.7E-05 |  |
| EXTL3 | chr8:28479635-28480798 | SSHELF | 0.030 | 4.6E-05 |  |
| **WNT10A** | **chr2:219762987-219763537** | **SSHELF** | **0.038** | **9.0E-05** | **SSHELF (boy)** |
| **DHX58** | **chr17:40250272-40250591** | **SSHELF** | **-0.061** | **3.1E-04** | **3-’UTR (boy)** |
| MT1F/G | chr16:56696892-56697293 | SSHORE | -0.025 | 9.0E-05 |  |
| RTN4RL2 | chr11:57243681-57244463 | SSHORE | 0.018 | 1.4E-04 |  |
| GSS | chr20:33543082-33543846 | SSHORE | 0.053 | 2.2E-04 |  |
| C7ORF50 | chr7:1062496-1062966 | SSHORE | 0.048 | 2.9E-04 |  |
| CHAT | chr10:50822350-50822666 | SSHORE | 0.021 | 3.1E-04 |  |

**Table S3**b: 450K analysis of girls group; regional top 5 hits

| **Girls** | | | | | | | |
| --- | --- | --- | --- | --- | --- | --- | --- |
| **Nearest Gene** | **Chr** | **Region** | **Delta** | **P-value** | **Overlap** | **Delta (T)** | **P-value (T)** |
| OR5M8 | 11 | Exon1 | 0.012 | 1.6E-04 | Exon1 (comb) | NA | |
| GIF | 11 | Exon1 | -0.030 | 3.9E-04 |  |
| WBP2 | 17 | Exon1 | 0.006 | 5.9E-04 |  |
| ZNF474 | 5 | Exon1 | 0.051 | 1.3E-03 |  |
| OLFML1 | 11 | Exon1 | -0.018 | 1.7E-03 |  |
| GABPB2 | 1 | Gene body | -0.017 | 4.6E-06 |  |
| HTR2A | 13 | Gene body | 0.069 | 8.1E-05 | Gene body (comb) |
| EIF4E2 | 2 | Gene body | 0.036 | 1.0E-04 | Gene body (comb) |
| PIAS2 | 18 | Gene body | -0.018 | 1.5E-04 |  |
| PSMA7 | 20 | Gene body | 0.013 | 3.7E-04 |  |
| ZXDB | X | TSS200 | 0.071 | 4.5E-04 |  |
| MTM1 | X | TSS200 | 0.032 | 7.0E-04 |  |
| C2CD4A | 15 | TSS200 | 0.006 | 8.5E-04 |  |
| MIR135B | 1 | TSS200 | -0.015 | 1.3E-03 |  |
| EIF3J | 15 | TSS200 | 0.006 | 1.3E-03 |  |
| SDCBP2 | 20 | TSS1500 | 0.087 | 5.8E-06 |  |
| ZNF366 | 5 | TSS1500 | 0.033 | 1.6E-04 | TSS1500 (comb) |
| UTS2R | 17 | TSS1500 | 0.067 | 1.7E-04 |  |
| PIGO | 9 | TSS1500 | 0.033 | 3.6E-04 |  |
| TWIST2 | 2 | TSS1500 | 0.013 | 5.1E-04 | TSS1500 (comb) |
| MICAL3 | 22 | 3-’UTR | 0.013 | 8.1E-05 |  |
| TRAPPC10 | 21 | 3-’UTR | -0.023 | 2.2E-04 |  |
| DULLARD | 17 | 3-’UTR | 0.017 | 3.1E-04 |  |
| HIAT1 | 1 | 3-’UTR | -0.036 | 3.8E-04 |  |
| NUMB | 14 | 3-’UTR | -0.049 | 5.2E-04 |  |
| GIF | 11 | 5-’UTR | -0.030 | 4.0E-04 |  |
| ZC3H14 | 14 | 5-’UTR | 0.068 | 4.5E-04 |  |
| WBP2 | 17 | 5-’UTR | 0.006 | 8.0E-04 |  |
| ZNF474 | 5 | 5-’UTR | 0.027 | 1.1E-03 |  |
| ACTR3C | 7 | 5-’UTR | 0.082 | 1.4E-03 |  |
| TWIST2 | chr2:239755096-239758310 | ISLAND | 0.013 | 3.5E-04 | TSS1500 (comb) |
| DPP6 | chr7:154684402-154684630 | ISLAND | 0.055 | 3.5E-04 |  |
| SCARF2 | chr22:20783466-20786201 | ISLAND | 0.039 | 5.9E-04 |  |
| TMEM121 | chr14:106025533-106026386 | ISLAND | -0.192 | 6.1E-04 |  |
| ARAF | chrX:47382821-47383584 | ISLAND | 0.046 | 6.8E-04 |  |
| AHNAK | chr11:62313282-62314314 | NSHELF | 0.041 | 9.2E-05 |  |
| GALR1 | chr18:76308229-76308578 | NSHELF | -0.044 | 1.9E-04 |  |
| PWP2 | chr21:45526956-45527813 | NSHELF | -0.023 | 2.5E-04 |  |
| ZNF175 | chr19:52074311-52074536 | NSHELF | -0.026 | 3.7E-04 |  |
| ST14 | chr11:130078173-130078667 | NSHELF | -0.029 | 4.3E-04 |  |
| FAM213A | chr10:82168064-82168917 | NSHORE | 0.042 | 1.8E-05 |  |
| PIAS2 | chr18:44496910-44497832 | NSHORE | -0.018 | 1.4E-04 |  |
| CENPJ | chr13:25496588-25497299 | NSHORE | 0.017 | 2.6E-04 |  |
| PSMA7 | chr20:60717598-60719667 | NSHORE | 0.024 | 4.2E-04 |  |
| FUBP1 | chr1:78444303-78444605 | NSHORE | 0.036 | 4.9E-04 |  |
| OSBP2 | chr22:31218226-31218893 | SSHELF | -0.015 | 7.4E-04 |  |
| STOX2 | chr4:184826253-184828177 | SSHELF | -0.020 | 7.7E-04 |  |
| USF2 | chr19:35760457-35760865 | SSHELF | 0.023 | 8.2E-04 |  |
| CACNA1B | chr9:141014643-141014870 | SSHELF | 0.093 | 9.0E-04 |  |
| TXNRD1 | chr12:104609397-104610172 | SSHELF | 0.030 | 9.4E-04 |  |
| ASCL5 | chr1:201083458-201084567 | SSHORE | -0.025 | 8.5E-05 |  |
| EIF4E2 | chr2:233415005-233415811 | SSHORE | 0.042 | 1.7E-04 | Gene body (comb) |
| NEXN | chr1:78354381-78354673 | SSHORE | 0.013 | 2.1E-04 |  |
| DOT1L | chr19:2163632-2165603 | SSHORE | 0.068 | 2.2E-04 |  |
| PIGO | chr9:35096152-35096673 | SSHORE | 0.033 | 3.8E-04 |  |

**Table S3c**: 450K analysis of boys group; regional top 5 hits

| **Boys** | | | | | | | |
| --- | --- | --- | --- | --- | --- | --- | --- |
| **Nearest Gene #** | **Chr** | **Region** | **Delta** | **P-value** | **Overlap** | **Delta (T)** | **P-value (T)** |
| ZNF281 | 1 | Exon1 | 0.034 | 1.3E-06 | Exon1 (comb) | 0.002 | 9.52E-01 |
| NAV3 | 12 | Exon1 | 0.018 | 1.1E-05 |  | -0.002 | 7.33E-01 |
| KIAA1324L | 7 | Exon1 | 0.016 | 3.2E-04 | Exon1 (comb) | -0.001 | 9.19E-01 |
| ZNF259 | 11 | Exon1 | 0.019 | 4.6E-04 |  | -0.021 | 1.84E-01 |
| OR1N1 | 9 | Exon1 | 0.061 | 5.0E-04 |  | 0.005 | 8.15E-01 |
| HOXC6 | 12 | Gene body | 0.011 | 3.6E-04 | Gene body (comb) | -0.001 | 8.68E-01 |
| LOC284233 | NA | Gene body | 0.038 | 4.3E-04 |  | -0.001 | 9.40E-01 |
| MAP7D3 | X | Gene body | 0.021 | 4.9E-04 |  | 0.003 | 2.36E-01 |
| SNORD116-14 | 15 | Gene body | -0.027 | 5.9E-04 |  | -0.003 | 8.36E-01 |
| C6orf164 | 6 | Gene body | 0.098 | 6.8E-04 |  | 0.027 | 3.50E-01 |
| DNAJC21 | 5 | TSS200 | 0.005 | 1.3E-04 |  | -0.001 | 6.95E-01 |
| ONECUT2 | 18 | TSS200 | 0.023 | 2.6E-04 |  | -0.011 | 4.35E-01 |
| OR5T1 | 11 | TSS200 | 0.044 | 3.9E-04 |  | 0.004 | 7.88E-01 |
| TRADD | 16 | TSS200 | 0.009 | 4.7E-04 |  | -0.001 | 8.37E-01 |
| BTNL8 | 5 | TSS200 | -0.013 | 7.3E-04 |  | -0.008 | 1.71E-01 |
| THAP3 | 1 | TSS1500 | 0.064 | 1.4E-05 |  | 0.032 | 4.54E-02 |
| SMG5 | 1 | TSS1500 | 0.046 | 3.3E-04 |  | 0.006 | 6.03E-01 |
| ZNF559 | 19 | TSS1500 | 0.040 | 3.4E-04 |  | -0.012 | 2.25E-01 |
| NMNAT3 | 3 | TSS1500 | 0.186 | 3.8E-04 |  | 0.067 | 3.44E-01 |
| TMEM184C | 4 | TSS1500 | 0.019 | 5.9E-04 |  | 0.006 | 6.49E-01 |
| DHX58 | 17 | 3-’UTR | -0.085 | 3.3E-04 | 3-’UTR (comb) | -0.009 | 6.37E-01 |
| USP7 | 16 | 3-’UTR | 0.024 | 3.7E-04 |  | 0.004 | 3.84E-01 |
| PRB4 | 12 | 3-’UTR | -0.015 | 5.2E-04 | 3-’UTR (comb) | 0.005 | 4.78E-01 |
| C17orf56 | 17 | 3-’UTR | 0.073 | 5.4E-04 |  | 0.007 | 7.81E-01 |
| CHL1 | 3 | 3-’UTR | 0.016 | 5.8E-04 |  | 0.018 | 1.36E-01 |
| ZNF281 | 1 | 5-’UTR | 0.017 | 1.5E-05 | Exon1 (comb) | 0.002 | 5.89E-01 |
| GIPC2 | 1 | 5-’UTR | 0.014 | 4.3E-04 |  | -0.008 | 1.68E-01 |
| TATDN3 | 1 | 5-’UTR | 0.007 | 5.2E-04 | 5-’UTR (comb) | 0.000 | 9.88E-01 |
| KCNA4 | 11 | 5-’UTR | 0.026 | 6.3E-04 |  | 0.007 | 5.26E-01 |
| TPRA1 | 3 | 5-’UTR | 0.025 | 1.1E-03 |  | -0.004 | 7.89E-01 |
| HOXC6 | chr12:54423427-54423712 | ISLAND | 0.011 | 1.5E-04 | Gene body (comb) | -0.001 | 8.62E-01 |
| RTN4R | chr22:20228961-20230275 | ISLAND | -0.018 | 2.0E-04 |  | 0.003 | 5.61E-01 |
| DNM1 | chr9:130996210-130996443 | ISLAND | -0.028 | 2.4E-04 | ISLAND (comb) | -0.017 | 8.10E-02 |
| COX6B2 | chr19:55864130-55864379 | ISLAND | 0.051 | 8.4E-04 |  | 0.020 | 1.45E-01 |
| BTNL9 | chr5:180486154-180486892 | ISLAND | 0.014 | 9.5E-04 |  | 0.007 | 2.99E-01 |
| FAM20C | chr7:218309-218951 | NSHELF | -0.018 | 3.2E-04 |  | 0.004 | 5.61E-01 |
| ARHGAP22 | chr10:49863620-49864601 | NSHELF | -0.018 | 5.3E-04 |  | -0.006 | 2.72E-01 |
| POLD4 | chr11:67120856-67121228 | NSHELF | 0.040 | 7.4E-04 |  | 0.004 | 7.81E-01 |
| HSPA4 | chr5:132387100-132388369 | NSHELF | -0.014 | 7.9E-04 |  | -0.011 | 4.01E-02 |
| MEOX1 | chr17:41791110-41791476 | NSHELF | 0.019 | 9.4E-04 |  | 0.002 | 8.30E-01 |
| NA | chr1:149224358-149224642 | NSHORE | 0.021 | 2.9E-05 |  | -0.004 | 4.41E-01 |
| PRDM5 | chr4:121843148-121844193 | NSHORE | 0.061 | 9.5E-05 |  | 0.025 | 1.10E-01 |
| MAP7D3 | chrX:135332893-135333726 | NSHORE | 0.040 | 1.4E-04 |  | NA | NA |
| LEPRE1 | chr1:43232214-43233359 | NSHORE | 0.123 | 1.6E-04 |  | 0.036 | 6.86E-02 |
| C16ORF80 | chr16:58162754-58163682 | NSHORE | 0.063 | 1.8E-04 |  | 0.005 | 6.98E-01 |
| ACHE | chr7:100492217-100494941 | SSHELF | -0.015 | 8.5E-05 |  | 0.013 | 5.54E-02 |
| C22ORF34 | chr22:50064655-50064984 | SSHELF | 0.045 | 1.9E-04 |  | 0.007 | 5.88E-01 |
| DHX58 | chr17:40250272-40250591 | SSHELF | -0.085 | 3.4E-04 | 3-’UTR (comb) | -0.009 | 6.38E-01 |
| WNT10A | chr2:219762987-219763537 | SSHELF | 0.039 | 4.1E-04 | SSHELF (comb) | 0.014 | 3.79E-01 |
| DZIP1L | chr3:137833848-137834592 | SSHELF | 0.028 | 4.6E-04 |  | -0.025 | 7.51E-02 |
| MYO10 | chr5:16935555-16936408 | SSHORE | -0.047 | 1.3E-04 |  | -0.011 | 4.41E-01 |
| LOC728554 | chr5:177302423-177302879 | SSHORE | 0.019 | 3.0E-04 |  | 0.002 | 7.21E-01 |
| A4GALT | chr22:43165947-43166287 | SSHORE | 0.026 | 3.3E-04 |  | 0.005 | 6.53E-01 |
| LOC100130093 | chr1:227916425-227916736 | SSHORE | 0.012 | 4.1E-04 |  | 0.001 | 7.50E-01 |
| COX6C | chr8:100905657-100906003 | SSHORE | 0.038 | 4.5E-04 |  | 0.012 | 3.68E-01 |

***Supplemental Tables S3a/b/c:*** # Nearest gene; For genomic regions concerning ISLAND, NSHORE, SSHORE, NSHELF and SSHELF, UCSC browser was used to determine the nearest gene, Chr; chromosome, Comb; combined analysis. (T); Tolerant, analysis result of former CMA patients vs. controls, cases were tolerant for cow’s milk at date of sampling. Bold; Epigenetic top 5 region overlapping between analyses with an absolute delta difference ≥ 0.01 between cases and controls, gene details described in main text.

**Table S4a**: Enrichment analysis combined

| **GO annotation** | **alias** | **class** | **P-value** |
| --- | --- | --- | --- |
| GO:1900245 | positive regulation of MDA-5 signaling pathway | 1 | 1,09E-04 |
| GO:0039533 | regulation of MDA-5 signaling pathway | 1 | 1,30E-04 |
| GO:0039530 | MDA-5 signaling pathway | 1 | 1,36E-04 |
| GO:1900246 | positive regulation of RIG-I signaling pathway | 1 | 1,48E-04 |
| GO:0039536 | negative regulation of RIG-I signaling pathway | 1 | 1,55E-04 |
| GO:1900758 | negative regulation of D-amino-acid oxidase activity | 0 | 1,72E-04 |
| GO:0030261 | chromosome condensation | 0 | 9,02E-04 |
| GO:0036289 | peptidyl-serine autophosphorylation | 0 | 1,31E-03 |
| GO:0048515 | spermatid differentiation | 0 | 2,05E-03 |
| GO:0072229 | metanephric proximal convoluted tubule development | 0 | 2,23E-03 |
| GO:0045824 | negative regulation of innate immune response | 1 | 2,34E-03 |
| GO:0001895 | retina homeostasis | 0 | 2,70E-03 |
| GO:0045008 | depyrimidination | 0 | 3,03E-03 |
| GO:0071474 | cellular hyperosmotic response | 0 | 3,19E-03 |
| GO:0061436 | establishment of skin barrier | 2 | 3,23E-03 |
| GO:0006610 | ribosomal protein import into nucleus | 0 | 3,25E-03 |
| GO:0032530 | regulation of microvillus organization | 0 | 3,26E-03 |
| GO:0035561 | regulation of chromatin binding | 0 | 3,36E-03 |
| GO:0035562 | negative regulation of chromatin binding | 0 | 3,43E-03 |
| GO:0009223 | pyrimidine deoxyribonucleotide catabolic process | 0 | 3,65E-03 |
| GO:0009992 | cellular water homeostasis | 0 | 3,70E-03 |
| GO:0032534 | regulation of microvillus assembly | 0 | 4,00E-03 |
| GO:0045773 | positive regulation of axon extension | 0 | 4,13E-03 |
| GO:0061144 | alveolar secondary septum development | 3 | 4,16E-03 |
| GO:0036159 | inner dynein arm assembly | 0 | 4,19E-03 |
| GO:0042985 | negative regulation of amyloid precursor protein biosynthetic process | 0 | 4,56E-03 |
| GO:0030240 | skeletal muscle thin filament assembly | 0 | 4,74E-03 |
| GO:0045198 | establishment of epithelial cell apical/basal polarity | 2 | 4,75E-03 |
| GO:0006244 | pyrimidine nucleotide catabolic process | 0 | 5,30E-03 |
| GO:0042984 | regulation of amyloid precursor protein biosynthetic process | 0 | 5,46E-03 |
| GO:0001969 | regulation of activation of membrane attack complex | 1 | 5,50E-03 |
| GO:0032929 | negative regulation of superoxide anion generation | 0 | 5,57E-03 |
| GO:0006323 | DNA packaging | 0 | 5,66E-03 |
| GO:0010561 | negative regulation of glycoprotein biosynthetic process | 0 | 5,71E-03 |
| GO:0031223 | auditory behavior | 0 | 5,86E-03 |
| GO:2000257 | regulation of protein activation cascade | 0 | 6,20E-03 |
| GO:0042297 | vocal learning | 0 | 6,48E-03 |
| GO:0030516 | regulation of axon extension | 0 | 6,77E-03 |
| GO:0045066 | regulatory T cell differentiation | 1 | 7,58E-03 |
| GO:0045075 | regulation of interleukin-12 biosynthetic process | 1 | 7,64E-03 |
| GO:0071816 | tail-anchored membrane protein insertion into ER membrane | 0 | 8,14E-03 |
| GO:0045589 | regulation of regulatory T cell differentiation | 1 | 8,23E-03 |
| GO:0044108 | cellular alcohol biosynthetic process | 0 | 8,33E-03 |
| GO:0071470 | cellular response to osmotic stress | 0 | 8,42E-03 |
| GO:0055107 | Golgi to secretory granule transport | 0 | 8,75E-03 |
| GO:0043482 | cellular pigment accumulation | 0 | 8,93E-03 |
| GO:0051041 | positive regulation of calcium-independent cell-cell adhesion | 0 | 9,22E-03 |
| GO:0006285 | base-excision repair, AP site formation | 0 | 9,52E-03 |
| GO:0035089 | establishment of apical/basal cell polarity | 0 | 9,69E-03 |
| GO:0006564 | L-serine biosynthetic process | 0 | 9,92E-03 |

**Table S4**b: Enrichment analysis girls

| **GO annotation** | **alias** | **class** | **P-value** |
| --- | --- | --- | --- |
| GO:0046005 | positive regulation of circadian sleep/wake cycle, REM sleep | 0 | 1,08E-03 |
| GO:0038032 | termination of G-protein coupled receptor signaling pathway | 0 | 1,29E-03 |
| GO:0023021 | termination of signal transduction | 0 | 1,34E-03 |
| GO:0042796 | snRNA transcription from RNA polymerase III promoter | 0 | 2,64E-03 |
| GO:0006042 | glucosamine biosynthetic process | 0 | 2,74E-03 |
| GO:0000966 | RNA 5'-end processing | 0 | 4,79E-03 |
| GO:0007338 | single fertilization | 0 | 5,02E-03 |
| GO:0071877 | regulation of adrenergic receptor signaling pathway | 0 | 5,16E-03 |
| GO:1901253 | negative regulation of egress of virus within host cell | 1 | 5,95E-03 |
| GO:0032859 | activation of Ral GTPase activity | 0 | 6,40E-03 |
| GO:0051807 | evasion or tolerance of defense response of other organism involved in symbiotic interaction | 1 | 6,44E-03 |
| GO:0006436 | tryptophanyl-tRNA aminoacylation | 0 | 7,07E-03 |
| GO:0090336 | positive regulation of brown fat cell differentiation | 0 | 7,43E-03 |
| GO:0051684 | maintenance of Golgi location | 0 | 7,52E-03 |
| GO:0090362 | positive regulation of platelet-derived growth factor production | 0 | 8,13E-03 |
| GO:0042746 | circadian sleep/wake cycle, wakefulness | 0 | 9,21E-03 |
| GO:0009566 | fertilization | 0 | 9,27E-03 |
| GO:0044068 | modulation by symbiont of host cellular process | 1 | 9,55E-03 |
| GO:0045075 | regulation of interleukin-12 biosynthetic process | 1 | 9,94E-03 |
| GO:0048515 | spermatid differentiation | 0 | 9,98E-03 |

**Table S4c**: Enrichment analysis boys

| **GO annotation** | **alias** | **class** | **P-value** |
| --- | --- | --- | --- |
| GO:0035308 | negative regulation of protein dephosphorylation | 0 | 1,51E-03 |
| GO:0043587 | tongue morphogenesis | 0 | 3,55E-03 |
| GO:0035305 | negative regulation of dephosphorylation | 0 | 4,26E-03 |
| GO:0050884 | neuromuscular process controlling posture | 0 | 4,33E-03 |
| GO:0016344 | meiotic chromosome movement towards spindle pole | 0 | 5,43E-03 |
| GO:0045198 | establishment of epithelial cell apical/basal polarity | 1 | 5,57E-03 |
| GO:0070649 | formin-nucleated actin cable assembly | 0 | 5,60E-03 |
| GO:1900245 | positive regulation of MDA-5 signaling pathway | 1 | 5,65E-03 |
| GO:0007132 | meiotic metaphase I | 0 | 5,72E-03 |
| GO:0061436 | establishment of skin barrier | 2 | 5,81E-03 |
| GO:0051295 | establishment of meiotic spindle localization | 0 | 5,82E-03 |
| GO:1901639 | XDP catabolic process | 0 | 5,88E-03 |
| GO:0039533 | regulation of MDA-5 signaling pathway | 1 | 6,31E-03 |
| GO:0045132 | meiotic chromosome segregation | 0 | 6,74E-03 |
| GO:0039536 | negative regulation of RIG-I signaling pathway | 1 | 6,94E-03 |
| GO:0043181 | vacuolar sequestering | 0 | 7,28E-03 |
| GO:0005984 | disaccharide metabolic process | 0 | 7,37E-03 |
